# Transvenous Lead Extraction in Adult Patient with Leads Implanted in Childhood-Is That the Same Procedure as in Other Adult Patients?

**DOI:** 10.3390/ijerph192114594

**Published:** 2022-11-07

**Authors:** Andrzej Kutarski, Wojciech Jacheć, Anna Polewczyk, Dorota Nowosielecka, Maria Miszczak-Knecht, Monika Brzezinska, Katarzyna Bieganowska

**Affiliations:** 1 Department of Cardiology, Medical University, 20-059 Lublin, Poland; 2 2nd Department of Cardiology, Faculty of Medical Sciences in Zabrze, Silesian Medical University, 41-800 Katowice, Poland; 3 Department of Physiology, Patophysiology and Clinical Immunology, Institute of Medical Sciences, Jan Kochanowski University, 25-369 Kielce, Poland; 4 Department of Cardiac Surgery, Świętokrzyskie Center of Cardiology, 25-736 Kielce, Poland; 5 Department of Cardiology, The Pope John Paul II Province Hospital, 22-400 Zamość, Poland; 6 Department of Cardiac Surgery, The Pope John Paul II Province Hospital, 22-400 Zamość, Poland; 7 Department of Cardiology, The Children’s Memorial Health Institute, 04-730 Warsaw, Poland

**Keywords:** pacing in children, lead extraction in young adults, complications of pacing, lead extraction complexity

## Abstract

Background: Lead management in children and young adults is still a matter of debate. Methods: To assess the course of transvenous lead extraction (TLE) in adults with pacemakers implanted in childhood (CIP) we compared 98 CIP patients with a control group consisting of adults with pacemakers implanted in adulthood (AIP). Results: CIP patients differed from AIP patients with respect to indications for TLE and pacing history. CIP patients were four–eight times more likely to require second-line or advanced tools. Furthermore, CIP patients more often than AIP were prone to developing complications: major complications (MC) (any) 2.6 times; hemopericardium 3.2 times; severe tricuspid valve damage 4.4 times; need for rescue cardiac surgery 3.7 times. The rate of procedural success was 11% lower because of 4.8 times more common lead remnants and 3.1 times more frequent permanently disabling complications. Conclusions: Due to system-related risk factors TLE in CIP patients is more difficult and complex. TLE in CIP is associated with an increased risk of MC and incomplete lead removal. A conservative strategy of lead management, acceptable in very old patients seems to be less suitable in CIP because it creates a subpopulation of patients at high risk of major complications during TLE in the future.

## 1. Introduction

Most children requiring permanent cardiac pacing receive an endocardial lead system [1,2]. Enthusiasm for the use of intracardiac pacing in children was not dampened by numerous reports on shorter function of intracardiac leads, mainly due to children’s natural activity and somatic growth [3,4,5,6,7]. Lead replacement as the optimal treatment strategy for children with lead dysfunction is recommended both in previous and recent guidelines [8,9,10]. However, its implementation in pediatric electrocardiology departments may differ. The PACELEAD study [11] provided a great deal of information on everyday practice. The survey examined the application of class IIa and IIb indications for lead extraction recommended in the 2009 HRS guidelines [8]. Lead extraction was preferred for class 2a indications by >70% of responders whereas lead abandonment was favored for 2b indications by >70% of respondents. The survey showed a tendency toward lead abandonment over lead extraction, especially in complex cases. The conclusion was that non-functional, dysfunctional, recalled, potentially harmful, or additional endocardial leads in pediatric patients, and patients with congenital heart disease posed significant challenges to physicians caring for those patients [8].

A strategy of lead abandonment may lead to a situation where a large number of children enter adulthood with old or very old leads, if still “functional”. Adequate pacing/sensing thresholds and normal impedance levels do not guarantee long lead function in the context of body growth [3,4,5,6,7]. All leads can be straightened, covered with a thick film of fibrous and usually calcified tissue, additionally promoting external lead insulation breaches. In our department (national reference center of children’s electrotherapy), non-functional leads, as a rule, have not been abandoned for the last 16 years. However, lead abandonment is a common strategy in other hospitals [11,12,13].

As a result, once children turn 18, they are referred to adult electrophysiologists. Limited lead lifetime, especially in children and young patients, creates the need for lead replacement or new lead implantation with abandonment of non-functional leads. Finally, most young adults with childhood-implanted endocardial pacing or/and ICD leads become candidates for lead extraction in adult centers.

To the best of our knowledge, there are fewer than 10 studies reporting the results of TLE in children and adolescents (i.e., ages 1–20, 25 and over) [4,5,14,15,16,17,18]. The investigators emphasize the distinctness of lead extraction in the population of such patients. This also accords with our earlier observations. Our experience shows that the issue of lead extraction in CIP patients is less known among physicians performing TLE in AIP patients. Furthermore, the studies conducted so far have not included comparisons of the effectiveness of TLE between CIP and AIP patients. This knowledge gap prompted us to undertake the present research.

The aim of the study was to define the distinctness and specificity of lead extraction in patients with childhood-implanted pacemakers (CIP) and adulthood-implanted pacemakers (AIP).

## 2. Methods

### 2.1. Study Population

This post hoc analysis used clinical data of 3344 patients who underwent transvenous lead extraction between March 2006 and September 2020. There were 2044 males and 1300 females ranging in age from 5 to 99, mean age 65.81 ± 15.6 years. All information relating to patients and procedures was entered into the computer on an ongoing basis. For the purposes of analyses and comparisons, we extracted two groups from the cohort. The first group consisted of 98 young adults ranging in age from 19 to 57 (mean age 27.46 ± 6.77 years) who were <19 years of age at their first CIED implantation (CIP). The second, control group consisted of 2659 patients with adulthood-implanted pacemakers (AIP): 1685 males and 994 females, ranging in age from 40 to 80 (mean age 66.49 ± 9.38) at first CIED implantation and transvenous lead extraction. The AIP patients best represent “ordinary” adult candidates for lead extraction. Patients who were <18 years of age during TLE and with leads implanted between 18–40 years of age as well as individuals >80 years of age during TLE were excluded from the analysis (they were different groups with respect to lead extraction). No other patient exclusion criteria were used.

### 2.2. Lead Extraction Procedure

The procedures of transvenous lead extraction were defined according to the 2009 and 2017 HRS and 2018 EHRA guidelines [8,9,10].

Lead extraction procedures were performed using mechanical systems such as polypropylene Byrd dilator sheaths (Cook^®^ Medical, Leechburg, PA, USA), mainly via the implant vein. If technical difficulties arose, alternative venous approaches and/or additional tools such as Evolution (Cook^®^ Medical, Leechburg, PA, USA), TightRail (Spectranetix, Sunnyvale, CA, USA), lassos, and basket catheters were used. Laser-cutting sheaths were not used. In both groups, lead extraction was performed by a team consisting of the same experienced ext ractor, a second operator having experience with pacing therapy, and a cardiac surgeon. An anesthesiologist and echocardiographist were often, but not always, present during the procedure. Indications for TLE and type of periprocedural complications were defined according to the *2017 HRS Expert Consensus Statement on Cardiovascular Implantable Electronic Device Lead Management and Extraction* [9].

### 2.3. Definitions

TLE was defined as the procedure for removal of leads implanted for more than one year using appropriate, dedicated equipment. Procedural success was defined as the extraction of all targeted leads in their entirety during the TLE procedure, with no fragments left, no permanent adverse health consequences for the patient, and no procedure-related death.

Clinical success was defined as the extraction of all targeted leads, with the possibility of leaving a small portion of the lead (<4 cm long), without adversely affecting the overall procedure and permanent clinical consequences for the patient (only in the case of procedures for non-infectious indications). The occurrence of permanent bodily injury, significant damage to the tricuspid valve or, for example, stroke or death related to the procedure despite optimal treatment, have precluded clinical success.

Partial radiographic success was defined when a small portion of the lead (<4 cm long) was left in place without negative clinical consequences. Partial radiographic success precluded procedural success and complete clinical success in the case of infection [8,9,10].

The SAFETY TLE score was used to assess the risk for the occurrence of major complications related to TLE [19]. The score takes into account the following parameters: sum of dwell times of extracted leads (threshold value ≥ 16.5 years), hemoglobin level in the blood (threshold level < 11.5 g/dL), female gender, the number of previous CIED-related procedures and age below 30 years at first CIED implantation. The number of expected major complications in the two groups was determined using the SAFETY TLE score calculator, an online tool available at http://alamay2.linuxpl.info/kalkulator/ (accessed on 2 November 2022).

### 2.4. Statistical Analysis

The Shapiro-Wilk test showed that most continuous variables were normally distributed. Continuous variables with normal distribution are presented as mean ± SD and with non-normal distribution as median and IQR. The categorical variables are presented as number and percentage. The significance of differences between groups was determined using the nonparametric Chi^2^ test with Yates correction (categorical variables) or the unpaired Mann-Whitney U test (continuous variables), which was used due to disproportions resulting from the small size of the CIP group. A *p*-value of less than 0.05 was considered as statistically significant. Statistical analysis was performed with STATISTICA 13.1 PL software (StatSoft, Cracow, Poland). 

### 2.5. Approval of the Bioethics Committee

All patients gave their informed, written consent to undergo TLE and use anonymous data from their medical records, approved by the Bioethics Committee at the Regional Chamber of Physicians in Lublin no. 288/2018/KB/VII.

## 3. Results

There are two striking findings from this comparative analysis (Table 1).

The two groups vary considerably in size, showing that young people with leads implanted in childhood represent a very small subpopulation of patients referred to TLE [98 (2.93%) out of 3344 consecutive TLEs].

There are more women in the CIP group than in the AIP group. Comparison in brief: CIP: generally healthy subjects, predominantly congenital/postoperative etiology and mechanical lead damage (electrical failure) as an indication for TLE. AIP: predominantly IHD, cardiomyopathies, valvular heart disease and co-morbidities, and infections as an indication for TLE.

Looking at main goals of TLE, certain tendencies can be observed (Table 2).

CIP patients were more likely to undergo device upgrading, superfluous lead extraction, and complete device system removal, whereas AIP patients were prone to infections. CIP patients usually received the simplest DDD and VVI devices, whereas AIP patients had ICD-V, CRT-P, and CRT-D devices. In consequence, adult patients were significantly more likely to have ICD leads, single-coil ICD leads, dual-coil ICD leads, and CS leads before TLE. The two groups did not differ in the average number of leads in the system and in the heart before TLE.

On the other hand, the data show that lead extraction is a more difficult and complicated procedure in CIP patients. They had more risk factors for difficulty of the procedure and major complications (MC): leads located on both sides of the chest, redundant lead length in the heart, previous lead extraction, longer dwell time of oldest lead, and longer mean implant duration (per patient) before TLE. In brief, in the CIP group, the number of patients with abandoned lead(s) was almost twice as high. They had fewer ICD leads, twice as many leads implanted on the right side of the chest, and three times more leads on both sides of the chest. This group of younger patients was 2 times as likely to have a history of previous TLE and 3 times as likely to have redundant lead length in the heart. They had undergone 1.2 times more CIED-related procedures before TLE, and had nearly two times as long an implant duration (dwell time of oldest lead per patient and mean implant duration per patient) before TLE.

The organization of the procedure plays an important role in lead removal. It has no influence on the development of major complications, but it may facilitate subsequent treatment if the complications do occur, and ultimately prevent procedure-related death (Table 3).

TLE was performed in consecutive patients in both groups (selection was done retrospectively). Venue of the procedure (electrophysiology laboratory; cardiac surgery operating room), the role of cardiac surgeons (standby; not scrubbed), type of anesthesia (general anesthesia; local anesthesia + general sedation, analgesia), TEE monitoring (as mandatory standard; lack of TEE monitoring as a rule) were in general similar in both groups. However, CIP patients were more often operated on in the hybrid room and in the presence of a cardiac surgeon as a co-operator.

The following risk factors were significantly more common in CIP patients: use of alternative venous entry site (other than left subclavian approach), extraction of broken leads with excess slack, and extraction of abandoned leads. AIP patients were more likely to undergo extraction of ICD leads and CS leads. CIP patients were characterized by oldest extracted leads (169.0 vs. 75.00 months in AIP group), longer (per patient) extracted lead dwell time (156.0 vs. 72.96 months in AIP group), longer cumulative dwell time of extracted leads (sum of dwell times of extracted leads in years) (20.67 vs. 8.83 years in AIP group), and a higher number of SAFETY TLE score points indicating the higher risk of MC (the number of points 10.35 vs. 5.65 in AIP group). The most important finding was that procedure-related risk factors were much more common and implant duration was twice as long in CIP than in AIP patients. The SAFETY TLE score indicated twice as high risk of MC in CIP patients.

***Procedure complexity***. According to the main goal of the study, most analyses were dedicated to complexity and technical problems during the procedure (Table 4).

Procedure duration is a valuable indicator of procedure complexity and difficulty. “Skin-to-skin time” (63.00 vs. 55.00 min), “sheath-to-sheath time” (21.00 vs. 8.00 min), and average time of single lead extraction (“sheath-to-sheath”/number of extracted leads) (12.50 vs. 4.50 min), were significantly longer in CIP patients. It means that in spite of simpler device systems, lead extraction was significantly more difficult and time-consuming in the CIP group.

The strategy of lead extraction was generally similar in both groups: never leave/abandon non-functional leads (with rare exceptions in seniors but never in young patients); replace “by the way” other functional but relatively old devices (PM > 10 years, ICD > 5 years to save all functional LV pacing or His bundle pacing leads). Extraction of abandoned leads was twice as common in CIP than in AIP patients. We analyzed unexpected procedure difficulties (UPD), or so-called technical problems, such as any obstruction in implant vein (subclavian region), lead-to-lead adhesions, Byrd dilator collapse/torsion/“fracture”, targeted lead fracture/rupture during extraction, and the need for using an alternative approach. Technical problems (UPD) were significantly more common in CIP patients. The occurrence of UPD forced the extractor to use additional tools such as metal sheaths, lasso catheters/snares, basket catheters, loops created with a catheter, guidewire and lasso, and an other-than-implant-vein approach.

In brief, in young patients, the procedure time was twice as long, the complexity of the procedure was higher, and unexpected difficulties during the procedure (technical problems) occurred 2.5–3 times more often, whereas the need for using second-line tools and advanced techniques was 4–8 times higher.

TLE outcomes encompass the occurrence of major complications, and rates of partial radiographic success, clinical success and procedural success. Non-graspable and non-removable lead remnants (<4 cm lead portion or tip of lead) were much more frequently observed in CIP than in AIP patients (9.5% and 6.6% vs. 1.6% and 1.9%) (Table 5).

**Major complications (MC)**. All major complications were more common in CIP rather than AIP patients. However, the two groups almost reached statistical significance in the need for rescue cardiac surgery (4.08% vs. 1.24%, *p* = 0.051). The remaining differences were not significant, but CIP and AIP patients clearly had a tendency to differ in relation to haemopericardium, hemothorax, and severe tricuspid valve damage. There were no procedure-related deaths (intra-, post-procedural) among CIP patients.

**Clinical success**. The rate of clinical success was similar in both groups (94.70% vs. 95.71%). The reasons for lack of clinical success were also comparable.

**Procedural success.** The rate of procedural success in CIP and AIP patients was significantly different (81.63% vs. 95.68%). The reasons for lack of procedural success were similar: lack of complete radiographic success (16.33% vs. 3.65%) and permanently disabling complications or death (2.04% vs. 0.68%). Tricuspid valve (TV) injury may be a major complication as a result of long implant duration and the extent of connective tissue response to long-term contact with heart structures. TV injury occurred more frequently in CIP patients: aggravation by 2 degrees (4.08% vs. 1.50%) and by 3 degrees (1.02% vs. 0.34%). In brief, all complications occurred more frequently in CIP than in AIP patients: major complications (any) 2.6 times; hemopericardium 3.2 times; severe tricuspid valve damage 4.4 times; the need for rescue cardiac surgery 3.7 times higher; and partial radiographic success 4.8 times more frequent. Clinical success was comparable but procedural success was 11% lower in CIP patients as they were more likely to have lead remnants and permanently disabling complications (4.6 times and 3.1 times, respectively).

## 4. Discussion

This paper addresses three questions: firstly, safety and efficacy of extraction in adults with leads implanted in childhood (CIP); secondly, difficulty and TLE-related risk in CIP patients and “ordinary” patients with leads implanted in adulthood (AIP), and thirdly, the mechanism by which a specific group of CIP patients is created in the context of the current guidelines and the findings from other original research papers.

We demonstrated that young adult patients with leads implanted in childhood (CIP) differed from “ordinary” patients with leads implanted in adulthood (AIP) with regard to indications for TLE, type of CIED, risk factors for MC, procedure difficulty, occurrence of UPD and MC, and rate of procedural success.

We selected 13 reports presenting over 1000 TLE procedures, and all 7 available reports on TLE in children, juveniles and young adults (Table 6). The table summarizes mean patient age, mean implant dwell time, major complications and procedure-related deaths. None of the large reports on TLE described a specific group of CIP patients. Such relatively rare patients were hidden among thousands of others, and there was no advancement of knowledge about this selected group of patients undergoing lead extraction. Mean age of adult patients was 65.2 years and average dwell time of extracted leads 74 months. In children and juveniles, the respective values were 18.4 years and 38.2 months. It means that in children and juveniles, lead extraction, if any, was performed 3 years earlier on average. Nevertheless, the rate of major complications was twice as high. Against this background (adults and children), our group of adult patients with leads implanted in childhood and TLE delayed as long as possible (mean age 27.2 years) is considerably different in terms of difficulty and safety of lead extraction. Mean implant duration of 172 months is 2.3 times longer than in the adult group and 4.5 times longer than in the pediatric and juvenile groups reported in the literature. The conclusion from this table is that a conservative lead management strategy and delay of lead replacement after serious complications have already arisen creates a group of patients in whom lead extraction becomes most difficult and risky.

**Figure 1:**
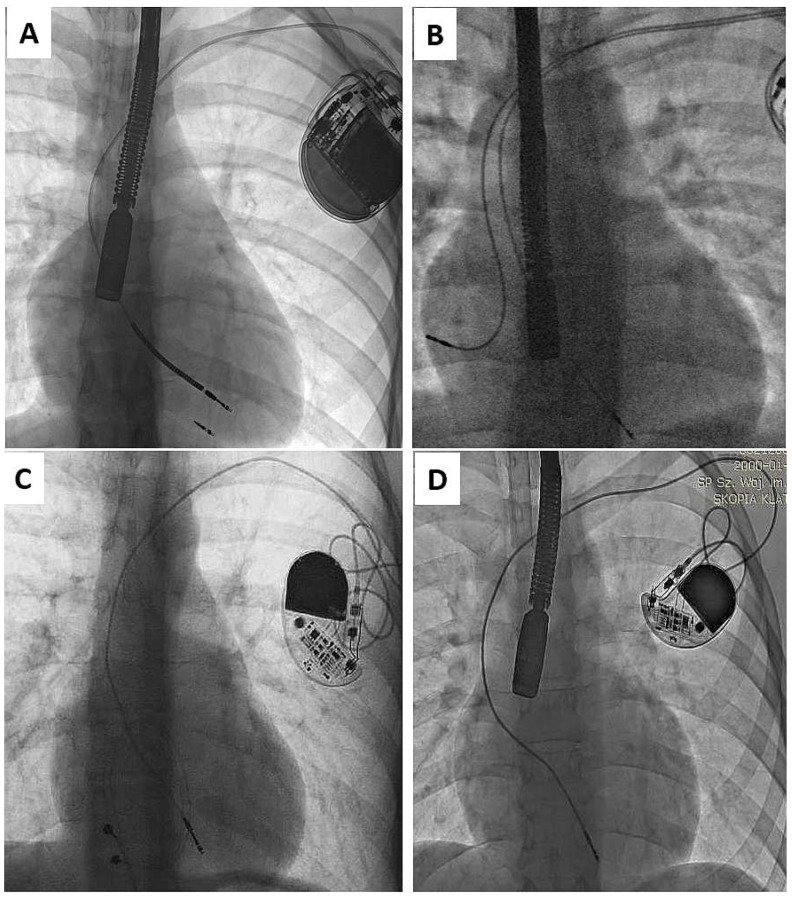
Straightening of the leads due to body growth (**A**–**D**). The unnatural course of the electrode is a common phenomenon in the analyzed population of patients. Adherence of the lead to the vein and heart structures induces scar formation and makes it difficult to extract the lead. Epicardial lead remnants (**C**) and endocardial led remnants (after previous TLE) (**A**) are frequent findings in patients with leads implanted in childhood. (**B**) strained RV lead, during unit replacement atrial lead in atypical tip position was added (**D**) straightening of the leads, no other pathology is seen.

**Figure 2:**
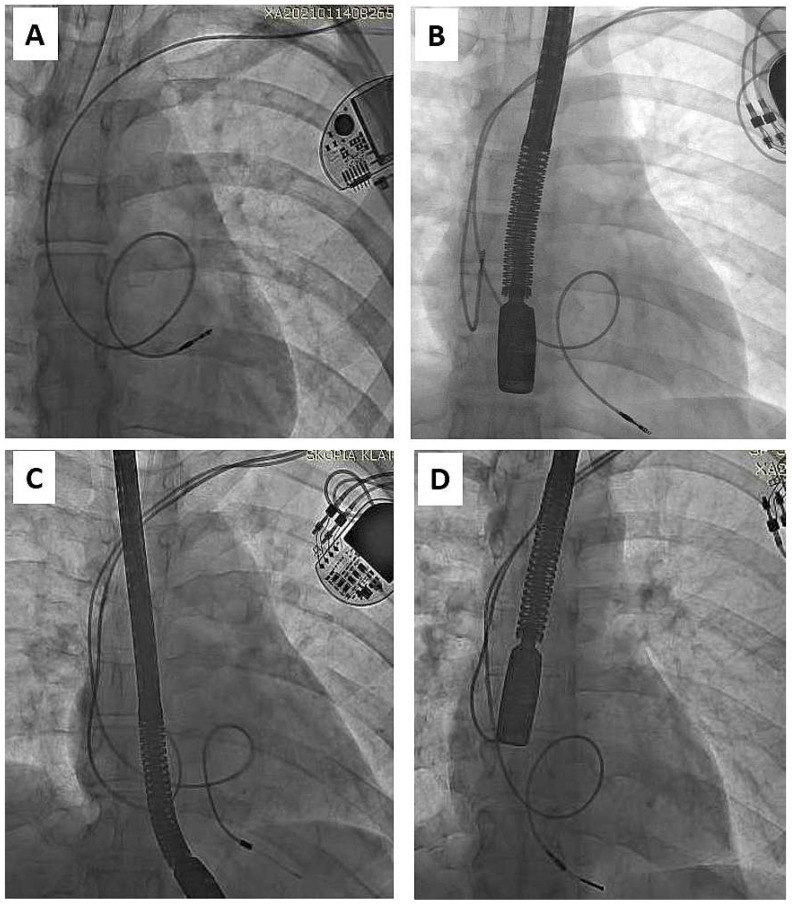
Another trap of lead extraction in young adults—redundant lead slack (but planned) resulting in strong adherence to the heart structures (examples **A**–**D**). It increases the risk of targeted lead fracture, atrial wall rupture or injury to the tricuspid apparatus. Lead slack to allow for growth was supposed to prevent lead straightening, but his technique did not live up to expectations and was abandoned. (**A**) VVI pacing system. Old unnecessary (but made on purpose) lead loop located in RA and RVOT. (**B**) DDD pacing system. Proper atrial lead route and loop on ventricular lead finally located in RVOT. (**C**) DDD pacing system two leads, two loops (**D**) Another one DDD system. Proper atrial lead course and loop of ventricular lead finally located in RVOT. Presence of old models of passive leads indicates for more difficult extraction.

As mentioned in the Introduction Section, the problem appears when children enter adulthood with old or very old leads, if still “functional”. Adequate pacing/sensing thresholds and normal impedance levels do not guarantee long lead function in the context of body growth [3,4,5,6,7,8,33]. Such functional leads, straightened (Figure 1) or looped (Figure 2), can adhere to the heart structures [34]. The leads are covered with a thick film of fibrous, usually calcified tissue, causing additional damage to external lead insulation (by pulling out and moving through the calcified scar). Limited lead longevity, especially in children and young patients [3,4,5,6,7,8,11,12,13,14,15,16,17,18,19] creates the need for lead replacement or new lead implantation with abandonment of non-functional leads [5,11,12,13].

**Figure 3:**
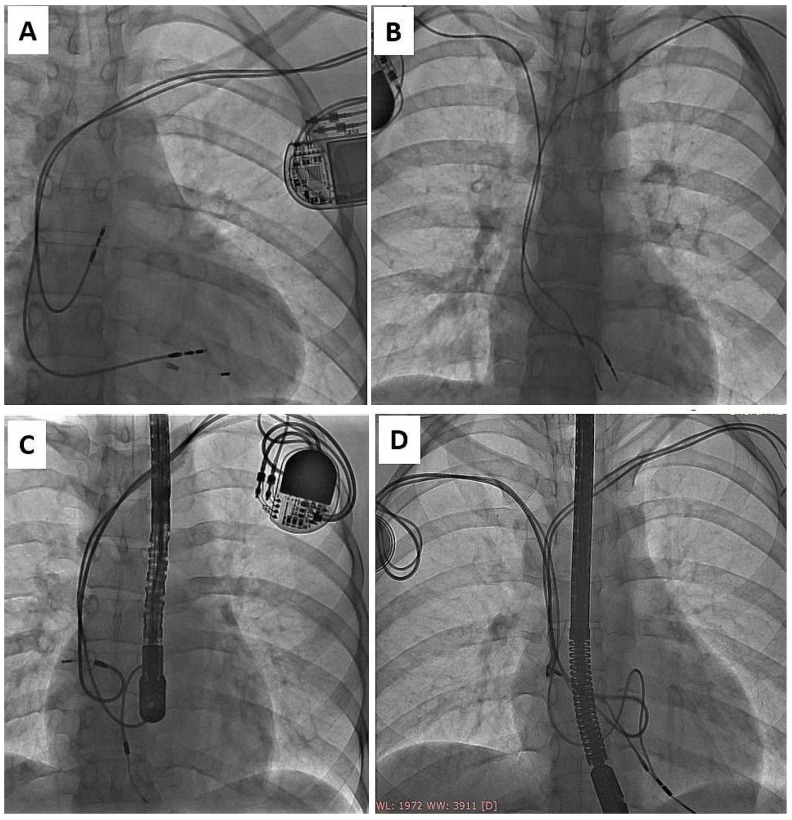
Common preoperative radiological imaging of adult patients with leads implanted in childhood, reflecting their history of electrotherapy in the form of remnants of lead fragments after TLE (**A**), abandoned leads (**B**,**D**) or unnecessary lead loops in the heart, which did not prevent constant pulling on the RV apex and partial destruction of BP passive lead (unnaturally increased tip-ring distance) (**C**). (**A**) Remnants of lead fragments after previous TLE. (**B**) Presence abandoned lead opposite chest side. (**C**) Two abnormal lead in right atrium, partial destruction of BP passive lead (unnaturally increased tip-ring distance). (**D**) Two abandoned leads left side of the chest. Redundant lead slack (but planned) generates risk of strong adherence to heart structures and to both functional leads and finally-very difficult extraction.

Finally, most young adults become candidates for lead extraction in adult centers or, receive new leads, and the next occasion for removal of all old leads is lead dysfunction or infection [33]. The adverse effects of lead abandonment in adults have been shown in several studies involving large patient populations [19,25,29,30,35]. Nobody has described abandoned lead-related problems in CIP patients, but our experience shows that a strategy of lead abandonment in children, juveniles, and young adults may create much more serious problems 10 to 20 years later (Figure 3).

As for the optimal time of lead extraction, several studies reflect a general tendency that it is better to wait until the body stops growing than to extract non-functional leads in pediatric patients [11,12]. We strongly disagree with this opinion.

The problem of prophylactic extraction of still functioning, but old, or very old, leads remains unsolved. During the past three decades, it has been demonstrated that the risk of lead removal doubles every three years and that lead durability is limited (longer for PM and shorter for ICD leads) [36,37,38]. There is still an open question about which strategy is better: prophylactic lead replacement after the end of the growth period or waiting until occurs in any old leads. Our experience shows that symptomatic patients with unexpected lead failure have a high chance of being admitted to their local hospitals where they usually receive an additional new lead without even considering lead replacement. This is the most frequent reason for lead abandonment and cause of challenging and risky lead extraction in the future (as described in our study). It should be remembered that delaying the decision to replace the leads at next unit revision is not only postponing the problem for another 10–12–14 years, but is also creating young patients with a lead implant duration of >20 years [38]. The conclusion is that a conservative lead management strategy and delay of lead replacement far into the future until the development of serious complications in children and in juveniles will ultimately result in building a group of individuals in whom lead extraction becomes most difficult and risky. The guidelines should be reviewed, taking into account the peculiarities of lead management in patients with leads implanted in childhood (CIP).

## 5. Study Limitations

This study has some limitations. The first limitation is the inclusion of only those patients who underwent transvenous lead extraction; there was no possibility of evaluating the entire CIP population.

Next, a relatively small size of the CIP group may affect the statistics, leading to an underestimation of statistical significance, but the proportion of the size of the test group to the control group represents the scale of the phenomenon.

The present study is a retrospective analysis, but patients were entered into the computer database immediately after TLE, regardless of treatment facility site. The organizational model of TLE procedures in other centers was not the same and changed over time. Only mechanical systems without laser energy devices were used for lead extraction. Finally, all extractions were performed by a single, very experienced first operator, in several centers and by various teams. It would not give the overview of general TLE safety and efficacy in children, and especially in young adults.

## 6. Conclusions

Despite overall good health, due to multiple system-related factors and much longer implant duration, TLE in young patients with leads implanted in childhood (CIP) is much more difficult and complicated than in older individuals. Due to multiple risk factors, TLE in CIP patients is associated with an increased risk of major complications and incomplete lead removal, and in consequence, a lower rate of procedural success. A conservative strategy of lead management, acceptable in old or very old patients (additional lead implantation and avoidance of TLE) seems to be less suitable in CIP patients because it creates a subpopulation of patients at high risk of major complications during TLE in the future.

## Figures and Tables

**Table 1:** Patient characteristics and indications for lead extraction.

Groups	CIP (A)		AIP (B)		A vs. B
Number	98		2659		
	Mean ± SD n (%)		Mean ± SD n (%)		*p*
**Patient characteristics**					
Patient age at TLE [years]	27.46	6.77	66.49	9.38	<0.001
Patient age at first system implantation [years]	13.04	4.47	58.44	11.51	<0.001
Sex (female patients)	48	48.98%	994	37.38	0.009
Etiology: (ischemic heart disease)	1	1.025%	1507	56.68	<0.001
Etiology: cardiomyopathy	5	5.10%	465	17.49	0.002
Etiology: congenital, channelopathy, neurocardiogenic, or cardiac surgery	92	93.88%	696	26.18	<0.001
NYHA class III and IV	0	0.00%	401	15.08	<0.001
LVEF average [%]	60.00	8.69	47.81	15.48	<0.001
Tricuspid regurgitation before TLE: significant	13	13.27%	362	13.61%	0.957
Tricuspid regurgitation before TLE: severe	3	3.06%	89	3.35%	0.895
Diabetes (any)	2	2.04%	561	21.10%	<0.001
Renal failure (any)	0	0.00%	543	20.42%	<0.001
Creatinine level [mg/dL]	0.83	0.17	1.24	1.84	<0.001
BMI [kg/m^2^]	24.34	4.40	28.31	5.40	<0.001
Previous sternotomy	20	21.04%	400	15.04%	0.191
Valve prosthesis	5	5.10%	216	8.12%	0.372
Long-term anticoagulation	8	8.16%	1076	40.47%	<0.001
Long-term antiplatelet treatment	7	7.14%	1229	46.22%	<0.001
Charlson comorbidity index [points]	0.20	1.01	4.73	3.52	<0.001
**Indications for TLE (main, predominant)**					
Systemic infection	12	12.24%	595	22.38%	0.024
Local (pocket) infection	6	6.12%	263	9.89%	0.289
Mechanical lead damage (electrical failure)	43	43.87%	684	25.72%	<0.001
Lead dysfunction (exit/entry block, dislodgement, or extracardiac pacing)	8	8.16%	331	12.45%	0.266
Lead dysfunction caused by (usually dry) perforation	6	6.12%	289	10.87%	0.185
Change of pacing mode/upgrading, downgrading	4	4.08%	163	6.13%	0.536
Abandoned lead/prevention of abandonment (AF, superfluous leads)	2	2.04%	87	3.27%	0.699
Threating/potentially threatening lead (loops, free ending, left heart, or LDTVD)	9	9.18%	80	3.01%	0.002
Other (MRI indications, cancer, painful pocket, or pacing/ICD no longer necessary)	5	5.10%	66	2.48%	0.199
Re-establishing venous access (symptomatic occlusion, SVC syndrome, or lead replacement/upgrading)	3	3.06%	101	3.80%	0.915

**Explanation of abbreviations**: CIP—childhood-implanted pacemakers (implantation at <19 years TLE at >19 years of age); AIP—adulthood-implanted pacemakers (implantation and TLE at 40–80 years of age); TLE—transvenous lead extraction; NYHA—New York Heart Association functional class; LVEF—left ventricular ejection fraction; BMI—body mass index; AF—atrial fibrillation; LDTVD—lead-dependent tricuspid valve dysfunction; MRI—magnetic resonance imaging; ICD—implantable cardioverter defibrillator; and SVC—superior vena cava.

**Table 2:** Main goals of lead extraction, system and history of pacing, leads before TLE.

Groups	CIP (A)		AIP (B)		A vs. B
Number	98		2659		
	Mean ± SD n (%) Median * IQR *		Mean ± SD n (%) Median * IQR *		*p*
**Patient/system/procedure information**					
System removal—infection	17	17.35%	779	29.30%	0.014
Upgrading	18	18.36%	294	11.06%	0.037
Downgrading	0	0.00%	100	3.76%	0.093
Lead replacement	48	48.97%	1289	48.48%	0.943
Superfluous lead extraction	7	7.14%	86	3.23%	0.069
Complete device system removal	4	4.08%	38	1.43%	0.092
System removal—reimplantation deferred	4	4.08%	73	2.75%	0.643
**System and history of pacing**					
PM—AAI	3	3.06%	192	7.22%	0.169
PM—DDD	62	63.27%	1185	44.57%	<0.001
PM—VDD	2	2.04%	53	1.99%	0.738
PM—VVI	22	22.45%	268	10.08%	<0.001
PM—CRT-P	0	0.00%	74	2.78%	0.175
Abandoned only PM lead (unit removed earlier) before TLE	1	1.02%	20	0.75%	0.771
ICD—VVI	2	2.04%	347	13.05%	0.002
ICD—DDD	6	6.12%	291	10.94%	0.178
ICD—CRT-D	0	0.00%	220	8.27%	0.006
Abandoned only ICD lead (unit removed earlier) before TLE	0	0.00%	8	0.30%	0.680
**Leads before TLE**					
Number of leads in the system	1.69	0.48	1.83	0.65	0.105
Patients with abandoned leads	19	19.39%	308	11.58%	0.029
Number of abandoned leads	0.26	0.60	0.16	0.49	0.328
Patients with multiple abandoned leads	6	6.12%	102	3.84%	0.367
Number of leads in the heart	1.96	0.74	1.98	0.77	0.392
ICD lead presence	7	7.14%	839	31.55%	<0.001
One single-coil ICD lead	3	3.06%	344	12.94%	0.006
Dual-coil ICD lead	2	2.04%	443	16.66%	<0.001
CS lead presence	1	1.02%	481	18.09%	<0.001
Leads on the left side of the chest	83	84.69%	2512	94.47%	<0.001
Leads on the right side of the chest	5	5.10%	67	2.52%	0.201
Leads on both sides of the chest	10	10.20%	80	3.01%	<0.001
Previous TLE	10	10.20%	126	4.74%	0.027
Excessive lead slack on X ray	16	16.33%	137	5.15%	<0.001
Number of procedures before lead extraction	2.23	1.14	1.87	1.02	<0.001
Dwell time of oldest lead per patient [months]	169.0 *	109.0 *	75.96 *	81.96 *	<0.001
Mean lead implant duration (per patient) [months]	156.0 *	85.68 *	72.00 *	73.68 *	<0.001

**Explanation of abbreviations**: TLE—transvenous lead extraction; CIP—childhood-implanted pacemakers (implantation at <19 years TLE at >19 years of age); AIP—adulthood-implanted pacemakers (implantation and TLE at 40–80 years of age); AAI—single-chamber pacemaker with the tip of the lead in the right atrium; VVI—single-chamber pacemaker with the tip of the lead in the right ventricle; DDD—dual-chamber pacemakers; CRT-P—cardiac resynchronization therapy pacemakers; ICD-VVI ICD—implantable ventricular cardioverter defibrillator; ICD-DDD—implantable dual-chamber cardioverter defibrillator; CRTD—cardiac resynchronization therapy defibrillator; PM—pacemaker; ICD—implantable cardioverter defibrillator; TLE—transvenous lead extraction; CS—coronary sinus; and excessive lead slack on X ray—too long lead loop in the heart which crosses tricuspid or pulmonary valve disturbing their function and obstructing lead extraction, *—Median and IQR.

**Table 3:** Course of TLE procedure and risk factors for major complications and procedure complexity.

Groups	CIP (A)		AIP (B)		A vs. B
Number	98		2659		
	Mean ± SD n (%) Median * IQR *		Mean ± SD n (%) Median * IQR *		*p*
**Venue of the procedure**					
Electrophysiology laboratory	42	42.86%	1405	52.84%	0.066
Cardiac surgery operating room	18	18.37%	504	18.99%	0.989
Hybrid room	38	38.78%	750	28.21%	0.031
**The role of cardiac surgeon**					
Co-operator	51	52.04%	1262	47.46%	0.468
Standby	47	47,96%	1397	52.54%	0.468
**Type of anesthesia**					
General anesthesia	47	47.96%	1162	43.70%	0.465
Local anesthesia + general sedation, analgesia	51	52.04%	1497	56.30%	0.465
**TEE monitoring as mandatory standard (with rare exceptions) since 2015 y**					
Routine TEE in monitoring lead extraction	38	38.78%	1022	38.44%	0.970
Lack of TEE monitoring during TLE procedure as the rule	60	61.22%	1637	61.56%	0.970
**Procedure-related risk factors for major complications and increased procedure complexity**					
Number of extracted leads in one patient	1.84	0.93	1.67	0.77	0.328
One or two leads were extracted	89	90.82%	2345	88.19%	0.719
Three or more leads were extracted	9	9.18%	312	11.73%	0.562
Leads extracted from both sides of the chest during the same TLE	4	4.08%	33	1.24%	0.051
Approach—left (side of the chest)	79	80.61%	2508	94.32%	<0.001
Approach—right (side of the chest)	6	6.12%	48	1.81%	0.008
Approach—both (sides of the chest)	4	4.08%	19	0.71%	0.002
Approach—subclavian + femoral	3	3.06%	21	0.79%	0.002
Extraction of lead with endocardial excessive slack	12	12.24%	94	3.54%	<0.001
Extraction of broken lead with endocardial excessive slack	3	3.06%	67	2.52%	0.994
Extraction of abandoned lead(s) (any)	19	19.39%	288	10.83%	0.015
Extraction of abandoned lead(s) (per patient)	2.27	0.02	0.15	0.00	0.030
ICD lead extracted	7	7.14%	788	29.64%	<0.001
CS (LV pacing) lead extracted	0	0.00%	185	6.96%	0.013
Oldest extracted lead dwell time [months]	169.0 *	96.00 *	75.00 *	81.96 *	<0.001
Mean (per patient) extracted lead dwell time [months]	156.0 *	89.04 *	72.96 *	87.76 *	<0.001
Cumulative dwell time of extracted leads (sum of dwell times of extracted leads) [years]	20.67 *	15.00 *	8.83 *	12.25 *	<0.001
SAFeTY score of MC risk [20]—number of points	10.35	4.16	5.65	4.21	<0.001

**Explanation of abbreviations**: TLE—transvenous lead extraction; CIP—childhood-implanted pacemakers (implantation at <19 years TLE at >19 years of age); AIP—adulthood-implanted pacemakers (implantation and TLE at 40–80 years of age); TEE—transesophageal echocardiography; ICD—implantable cardioverter defibrillator; CS—coronary sinus, SAFETY—acronym, SAFETY TLE score; where: S = sum of lead dwell times, A = anemia, Fe = female, T = treatment (previous procedures), Y = young patients, and TLE = transvenous lead extraction, *—Median and IQR.

**Table 4:** Lead extraction procedure complexity.

Groups	CIP (A)		AIP (B)		A vs. B
Number	98		2659		
	Median * IQR * n (%)		Median * IQR * n (%)		*p*
**Procedure complexity**					
Procedure duration (“skin-to-skin”) [minutes]	63.00 *	22.00 *	55.00 *	20.00 *	<0.001
Procedure duration (“sheath-to-sheath”) [minutes]	21.00 *	26.00 *	8.00 *	8.00 *	<0.001
Mean time of single lead extraction (“sheath-to-sheath”/number of extracted leads) [minutes] All leads extracted	12.50 *	18.00 *	4.50 *	5.00 *	<0.001
	84	88.42%	2001	75.25%	0.021
Functional leads left in place for continued use	14	14.74%	636	23.92%	<0.001
Non-functional leads left in place	0	0.00%	16	0.60%	0.926
Non-functional, superfluous leads extracted	19	20.00%	288	10.83%	0.013
**Procedure complexity** **/unexpected technical problems**					
Technical problem during TLE (any)	48	50.53%	521	19.59%	<0.001
Blockage in implant vein (subclavian region)	16	16.84%	188	7.07%	<0.001
Lead-to-lead adhesion	14	14.74%	181	6.81%	0.008
Byrd dilator collapse/torsion/“fracture”	15	15.79%	77	2.90%	<0.001
Extracted lead fracture/rupture during extraction	22	23.16%	150	5.64%	<0.001
Need to use alternative approach	14	14.74%	103	3.87%	<0.001
Number of technical problems	1.61	1.00	1.35	0.67	<0.001
One technical problem only	24	25.26%	304	11.43%	<0.001
Two technical problems	13	13.68%	74	2.78%	<0.001
Three or more technical problems	4	4.21%	30	1.13%	0.033
Other minor technical problems	14	14.74%	126	4.74%	<0.001
**Use of additional tools**					
Evolution (old and RL) or TightRail	9	9.47%	30	1.13%	<0.001
Metal sheaths	17	17.89%	183	6.88%	<0.001
Lasso catheters/snares/basket catheters	14	14.74%	86	3.23%	<0.001
Loop created with a catheter, guidewire, and lasso	3	3.16%	47	1.77%	0.478

**Explanation of abbreviations**: TLE—transvenous lead extraction; CIP—childhood-implanted pacemakers (implantation at <19 years/TLE at >19 years of age); AIP—adulthood-implanted pacemakers (implantation and TLE at 40–80 years of age), *—Median and IQR

**Table 5:** Efficacy and complications of transvenous lead extraction.

Groups	CIP (A)		AIP (B)		A vs. B *p*
Number	98		2659		
**Partial or lack of radiographic success**					
Partial radiographic success (retained tip of lead)	6	6.12%	51	1.92%	0.012
Partial radiographic success (retained <4 cm lead fragment)	9	9.18%	42	1.58%	<0.001
Lack of radiographic success (retained lead or long portion of lead)	0	0.00%	5	0.19%	0.440
**Major complications**					
Major complications (any)	5	5.10%	54	2.03%	0.088
Hemopericardium	4	4.08%	35	1.32%	0.066
Hemothorax	0	0.00%	5	0.19%	0.667
Tricuspid valve injury during TLE (severe)	2	2.04%	13	0.49%	0.176
Rescue cardiac surgery	4	4.08%	33	1.24%	0.051
Minor complications (any)	12	12.24%	201	7.56%	0.130
Procedure-related death (intra-, post-procedural)	0	0.00%	6	0.23%	0.527
Indication-related death (intra-, post-procedural	0	0.00%	2	0.08%	0.101
**Clinical success**					
Clinical success	93	94.90%	2545	95.71%	0.891
No; planned supplementary TLE or cardiac surgery	0	0.00%	90	3.38%	0.118
No; complication—death	2	2.04%	17	0.64%	0.305
**Procedural success**					
Complete procedural success	80	81.63%	2544	95.68%	<0.001
No; lack of complete radiographic success	16	16.33%	97	3.65%	<0.001
No; permanently disabling complication or death	2	2.04%	18	0.68%	0.339
**Additional procedure information**					
Pacemaker dependence	35	35.71%	442	16.62%	<0.001
**Condition of extracted leads (intracardiac lead abrasion)**					
Probable abrasion (lead significantly damaged)	12	12.24%	77	2.90%	<0.001
Certain lead abrasion	30	30.61%	451	16.96%	<0.001
**TV injury during TLE**					
TR increase by 2 degrees	4	4.08%	40	1.50%	0.112
TR increase by 3 degrees	1	1.02%	9	0.34%	0.805

**Explanation of abbreviations**: TLE—transvenous lead extraction; CIP—childhood-implanted pacemakers (implantation at <19 years/TLE at >19 years of age); AIP—adulthood-implanted pacemakers (implantation and TLE at 40–80 years of age); TV—tricuspid valve; and TR—tricuspid regurgitation.

**Table 6:** Analysis of the literature concerning TLE. The comparison of mean patient age, mean implant dwell time, major complications, and procedure-related deaths in adults versus children and juveniles.

Reference Number	Year, Author, Journal	Number of Pts	Mean Age of Patients	Mean Dwell Time	Major Complications	Procedure-Related Death
**Studies in Adults (Reporting > 1000 TLE Procedures)**						
[20]	1999, Byrd, C.L., *Pacing Clin Electrophysiol*	2338	64	47	1.40%	0.40%
[21]	2008, Bongiorni, M., *Eur Heart J.*	1193	66	69	0.70%	0.30%
[22]	2010, Wazny, O., *J Am Coll Cardiol. LEXICon Sudy*	1449	63	82	1.40%	0.30%
[23]	2014, Brunner, M.P., *Heart Rhythm*	2999	67	61	1.80%	0.20%
[24]	2016, Bashir, J., *Circ Arrhythm Electrophysiol*	1082	59	129	3.00%	0.37%
[25]	2017, Hussein, A.A., *JACC Clin Electrophysiol*	1836	68	107.5	1.93%	0.29%
[26]	2017, Kutarski, A., *Europace*	2049	65	89	1.80%	0.36%
[27]	2017, Bongiorni, M., *Eur Heart Journal*	3555	65	76.8	1.70%	0.50%
[28]	2018, Sood, N., *Circ Arrhythm Electrophysiol*	11,304	65	65	2.30%	0.16%
[29]	2019, Jacheć, W., *Pacing Clin Electrophysiol*	3810	65	86.4	1.44%	0.17%
[30]	2020, Segreti, L., *Europace*	1210	69	72	0.70%	0.16%
[31]	2020, Starck, C.T., *Europace*	2205	66	74	1.00%	0.18%
[32]	2020, Giannotti Santoro, M., *Pacing Clin Electrophysiol*	1316	65	72	0.70%	0.00%
**All studies in adults, summary**		36,346	65.2	74.16	1.75%	0.24%
**Studies in children and juveniles (all available studies)**						
[14]	1996, Friedman, R.A., 1, *PacingClinElectrophysiol*	13	13.1	54	0.00%	0.00%
[6]	2003, Cooper, J.M., *J CardiovascElectrophysiol*	14	17.9	42.4	0.00%	0.00%
[15]	2006, Moak, J.P., *Pacing Clin Electrophysiol*	25	10	49.4	8.00%	0.00%
[16]	2009, Dilber, E., *Med Princ Pract*	30	12	46	2.80%	0.00%
[17]	2010, Cecchin, F., *Circ Arrhythm Electrophysiol*	144	21.5	86.8	2.80%	0.00%
[18]	2010, Zartner, P.A., *Europace*	22	12.9	61.2	0.00%	0.00%
[5]	2013, Atallah, J., *Circulation*	879	18.6	28.8	4.00%	0.00%
**Studies in children and juveniles, summary**		1127	18.42	38.22	3.73%	0.00%
**Our group of patients with leads implanted in childhood**		**98**	**27.5**	**171.8**	**5.26%**	**0.00%**
**Our control group of adult patients** **(40–80 years of age)**		2659	66.5	95.6	2.03%	0.23%

## Data Availability

Readers can access the data supporting the conclusions of the study at www.usuwanieelektrod.pl (accessed on 2 November 2022).
